# Prognostic biomarkers for the response to the radiosensitizer nimorazole combined with RCTx: a pre-clinical trial in HNSCC xenografts

**DOI:** 10.1186/s12967-023-04439-2

**Published:** 2023-08-26

**Authors:** Lydia Koi, Verena Bitto, Corina Weise, Lisa Möbius, Annett Linge, Steffen Löck, Ala Yaromina, María José Besso, Chiara Valentini, Manuel Pfeifer, Jens Overgaard, Daniel Zips, Ina Kurth, Mechthild Krause, Michael Baumann

**Affiliations:** 1 OncoRay – National Center for Radiation Research in Oncology, Faculty of Medicine, Helmholtz-Zentrum Dresden - Rossendorf, University Hospital Carl Gustav Carus, Technische Universität Dresden, Dresden, Germany; 2 Department of Radiotherapy and Radiation Oncology, Faculty of Medicine, University Hospital Carl Gustav Carus, Technische Universität Dresden, Dresden, Germany; 3 Helmholtz-Zentrum Dresden - Rossendorf, Institute of Radiooncology – OncoRay, Dresden, Germany; 4 Division of Applied Bioinformatics, German Cancer Research Center (DKFZ), Heidelberg, Germany; 5 Division of Radiooncology / Radiobiology, German Cancer Research Center (DKFZ), Heidelberg, Germany; 6 HIDSS4Health – Helmholtz Information and Data Science School for Health, Karlsruhe/Heidelberg, Germany; 7 German Cancer Consortium (DKTK), Partner Site Dresden, and German Cancer Research Center (DKFZ), Heidelberg, Germany; 8 National Center for Tumor Diseases (NCT), Partner Site Dresden, German Cancer Research Center (DKFZ), Heidelberg; Faculty of Medicine and University Hospital Carl Gustav Carus, Technische Universität Dresden, and Helmholtz-Zentrum Dresden - Rossendorf, Dresden, Germany; 9 The M-Lab, Department of Precision Medicine, GROW – School for Oncology and Reproduction, Maastricht University, Maastricht, The Netherlands; 10 Institute of Legal Medicine, Medizinische Fakultät, Technische Universität Dresden, Dresden, Germany; 11 Department of Radiation Oncology, University Hospital Aarhus, Aarhus, Denmark; 12 Corporate member of Freie Universität Berlin and Humboldt Universität Zu Berlin, Department of Radiation Oncology, Charité - Universitätsmedizin Berlin, Berlin, Germany

**Keywords:** HNSCC, Hypoxia, Radioresistance, Radiotherapy, Radiochemotherapy, Radiosensitizer, Nimorazole, Biomarker

## Abstract

**Background:**

Tumor hypoxia is associated with resistance to radiotherapy and chemotherapy. In head and neck squamous cell carcinoma (HNSCC), nimorazole, an oxygen mimic, combined with radiotherapy (RT) enabled to improve loco-regional control (LRC) in some patients with hypoxic tumors but it is unknown whether this holds also for radiochemotherapy (RCTx). Here, we investigated the impact of nimorazole combined with RCTx in HNSCC xenografts and explored molecular biomarkers for its targeted use.

**Methods:**

Irradiations were performed with 30 fractions in 6 weeks combined with weekly cisplatin. Nimorazole was applied before each fraction, beginning with the first or after ten fractions. Effect of RCTx with or without addition of nimorazole was quantified as permanent local control after irradiation. For histological evaluation and targeted gene expression analysis, tumors were excised untreated or after ten fractions. Using quantitative image analysis, micromilieu parameters were determined.

**Results:**

Nimorazole combined with RCTx significantly improved permanent local control in two tumor models, and showed a potential improvement in two additional models. In these four models, pimonidazole hypoxic volume (pHV) was significantly reduced after ten fractions of RCTx alone. Our results suggest that nimorazole combined with RCTx might improve TCR compared to RCTx alone if hypoxia is decreased during the course of RCTx but further experiments are warranted to verify this association. Differential gene expression analysis revealed 12 genes as potential for RCTx response. When evaluated in patients with HNSCC who were treated with primary RCTx, these genes were predictive for LRC.

**Conclusions:**

Nimorazole combined with RCTx improved local tumor control in some but not in all HNSCC xenografts. We identified prognostic biomarkers with the potential for translation to patients with HNSCC.

**Supplementary Information:**

The online version contains supplementary material available at 10.1186/s12967-023-04439-2.

## Background

It is known for a long time that well oxygenated tumor cells exhibit a higher sensitivity to X-rays compared to hypoxic cells, quantified by the oxygen enhancement ratio which ranges between 2.7 and 3.0 [1]. In pre-clinical and clinical studies, local tumor control rates after radiotherapy (RT) are lower in hypoxic head and neck squamous cell carcinoma (HNSCC) tumors compared to better oxygenated tumors [2–6], highlighting the need for hypoxia-related biomarkers. Yet, no gold standard to assess tumor hypoxia has evolved from the proposed ones, like hypoxia gene signatures, positron emission tomography (PET) imaging parameters or pimonidazole binding levels. Hypoxia gene signatures group patients into having either more or less hypoxic tumors based on expression levels of hypoxia-associated genes. For HNSCC, several hypoxia gene signatures with prognostic value for therapy outcome on various endpoints have been proposed [7–10]. Also, hypoxia estimation through pimonidazole binding in untreated tumor biopsies, measured as pimonidazole hypoxic fraction, has proven prognostic for loco-regional control (LRC) in patients with HNSCC [3]. In our previous experiments on HNSCC xenografts, we investigated additional micromilieu parameters besides pimonidazole hypoxic fraction before and during fractionated irradiation [5, 6, 11]. In these experiments, especially pimonidazole hypoxic volume and the fraction of perfused vessels after 10 fractions of RT have emerged as promising prognostic factors for tumor control [6]. Other strategies to obtain the hypoxic volume of a tumor include PET imaging approaches using either ^18^F-Fluoromisonidazole (FMISO), or ^18^F-Fluoroazomycin-arabinoside (FAZA) tracers [12–14], with further promising hypoxia tracers like ^18^F-Flortanidazole ([18F]-HX4) being under investigation [15]. For FMISO PET scans, residual hypoxia measured after two weeks during fractionated radiochemotherapy was prognostic for LRC [13, 14, 16], later complemented by further prognostic pre-treatment parameters for FMISO and FAZA [17]. On the interventional side, diverse strategies to overcome hypoxia-associated radioresistance have been investigated in clinical trials, such as oxygen breathing, mimicking of oxygen by means of nitroimidazoles and the selective killing of hypoxic cells, e.g. using tirapazamine [1]. Studies on 5-nitroimidazoles demonstrated that especially nimorazole (1-(N-β-ethylmorpholine)-5-nitro-imidazole) allows for clinical relevant radiosensitization of hypoxic cells, while being less toxic than 2-nitroimidazoles, e.g., misonidazole [18]. In Denmark, the addition of nimorazole to RT was studied in patients with HNSCC already in the 1990s (Danish Head and Neck Cancer Group [DAHANCA] 5 [19]), leading to significant enhancement of LRC compared to RT alone. Retrospectively, Toustrup et al. demonstrated that predominately patients with more hypoxic tumors, assessed via the hypoxia 15 gene signature, benefited from the addition of nimorazole [9]. Also, the human papilloma virus (HPV) infection status of patients was associated with the response to nimorazole, i.e., only patients with HPV-negative tumors showed an improved LRC. Later, accelerated fractionation [20] and additional chemotherapy [21] have been added to the combination of radiotherapy with nimorazole as next steps of treatment intensification. This has resulted in today’s unique standard of care for patients with non-operable HNSCC in Denmark which combines accelerated radiotherapy with nimorazole and weekly cisplatin [22], while in other countries radiotherapy with cisplatin has evolved as clinical standard. In a retrospective comparison of the two standards, involving DAHANCA patients from Denmark and Princess Margaret Hospital Cancer Centre (PMH) patients from Canada, comparable treatment outcomes were observed [23]. In that study, they also reasserted results from meta-analyses [24], confirming that concomitant chemotherapy to radiotherapy is an independent prognostic factor for LRC and overall survival. However, currently missing remains a study assessing the effectiveness of nimorazole to improve LRC when given in addition to radiotherapy combined with chemotherapy, i.e., radiochemotherapy (RCTx). Recently, the DAHANCA 29-EORTC 1219 (NCT01880359) trial aimed to evaluate the effect of nimorazole during accelerated RCTx but was closed early due to a weaker treatment effect as hypothesized [25]. Thus, the question if nimorazole is able to further improve LRC in RCTx regimes remains open. In this pre-clinical trial, we investigated if nimorazole combined with fractionated RCTx improves tumor control rate in HNSCC xenografts compared to RCTx alone and whether the effect of nimorazole is uniform in different tumor models. Additionally, we examined whether micromilieu parameters or gene expression profiles can be identified pre-treatment or during treatment that may serve as prognostic or predictive biomarker for treatment outcome. Promising candidate genes were tested for clinical relevance in human HNSCC.

## Methods

**Fig. 1:**
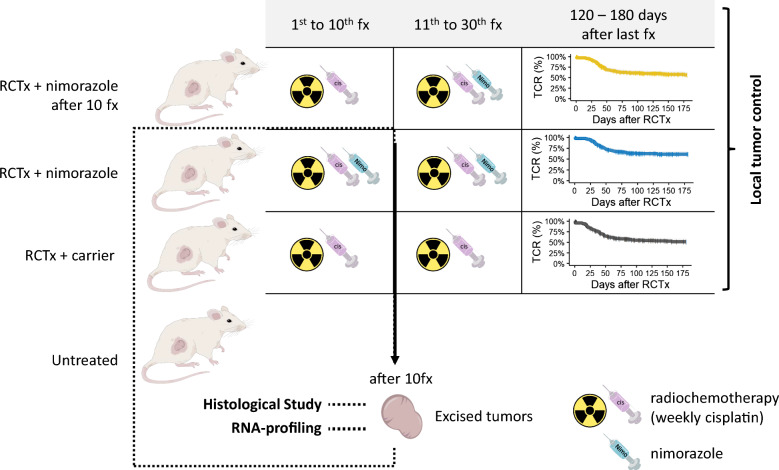
Experimental setup: For local tumor control we investigated radiochemotherapy (RCTx) plus weekly cislatin (cis) with and without nimorazole (nimo) using the following three treatment arms: RCTx + nimorazole after 10 fractions (fx), RCTx + nimorazole and RCTx + carrier. For histological evaluation and RNA-profiling we investigated RCTx + carrier and RCTx + nimorazole treatment after 10fx as well as untreated tumors. Abbreviations in graphic: nimo: nimorazole, cis: cisplatin

Local tumor control was evaluated in seven different HNSCC xenograft models and three treatment groups each, receiving 30 fractions of either RCTx (RCTx + carrier) or RCTx combined with nimorazole, starting nimorazole addition after ten fractions (RCTx + nimorazole after 10fx) or with the first fraction (RCTx + nimorazole). Biomarker discovery was carried out on xenograft models which remained untreated (Untreated) or received 10 fractions of either RCTx (10fx RCTx + carrier) or RCTx combined with nimorazole (10fx RCTx + nimorazole). The experimental setup is summarized in Fig. 1.

### Animals and tumor models

The animal facility and the experiments followed the ARRIVE guidelines and were approved according to the institutional guidelines and the German animal welfare regulations. The experiments were performed using 7–14 week-old male and female NMRI (nu/nu) mice obtained from the pathogen-free animal breeding facility (OncoRay—National Center for Radiation Research in Oncology, Faculty of Medicine and University Hospital Carl Gustav Carus, Technische Universität Dresden, Helmholtz-Zentrum Dresden—Rossendorf, Dresden, Germany). The experiments were performed using the HNSCC cell lines FaDu, SAS, UT-SCC-5 (UT5), UT-SCC-8 (UT8), CAL33, UT-SCC-45 (UT45) and SAT (Table 1 [26]), which have been previously described in detail [5, 27–29]. To immunosuppress the nude mice further, they received total body irradiation with 4 Gy (200 kV X-rays, 0.5 mm Cu-filter, ∼ 1 Gy/min) two to five days before tumor transplantation. Small pieces of tumors generated from a cryostock were transplanted subcutaneously into the right hind leg of anesthetized mice (120 mg/kg body weight [b.w.] ketamine intraperitoneal [i.p.] and 16 mg/kg xylazine i.p.). Histological examinations, DNA-microsatellite profile and volume doubling time confirmed the identity of all transplanted tumor xenografts. All inclusion and exclusion criteria were defined before the experiment and are stated in the following subsections.

**Table 1:** Characteristics of all head and neck squamous carcinoma (HNSCC) cell lines used in this pre-clinical study

Name	Sex	Age	Anatomical site	HPV status
FaDu	Male	56	Hypopharynx	HPV-negative
SAS	Female	69	Oral cavity (tongue)	HPV-negative
UT-SCC-5	Male	58	Oral cavity (tongue)	HPV-negative
UT-SCC-8	Male	42	Larynx	HPV-negative
CAL33	Male	69	Oral cavity (tongue)	HPV-negative
UT-SCC-45	Male	76	Oral cavity (floor of mouth)	HPV33-positive
SAT	Male	nd	Oral cavity	HPV-negative

### Nimorazole and cisplatin administration

Nimorazole, in the context of this research cooperation, was supplied by Department of Experimental Clinical Oncology, Aarhus University Hospital, Denmark (Prof. Jens Overgaard). In the experimental group, nimorazole was dissolved immediately before administration in sodium chloride (0.9%) to a concentration of 0.3 mg/g b.w. and was injected i.p. 30 min before each irradiation fraction at a volume of 0.01 ml/g b.w. [30]. Control animals were injected with the same volume of sodium chloride as carrier. Cisplatin (Calbiochem, Germany, 3 mg/kg b.w.) dissolved in sodium chloride (0.9%) was administrated i.p. at the first day of treatment and then once weekly directly before irradiation. The administered dose of nimorazole was chosen to be clinically relevant. The effectiveness of the same dose (0.3 mg/g) was verified in C3H mammary carcinoma mouse models previously [18], in which nimorazole in combination with fractionated RT produced a significantly enhanced radiation response compared to irradiation alone (enhancement ratio of 1.26).

### Local tumor irradiation and experimental design

Local irradiations were given with 200 kV X-rays (0.5 mm Cu-filter) at a dose rate of ~ 1 Gy/min; specially designed jigs were able to hold up to five animals at the same time. Based on previous results with RT alone [6, 27–29], radiation doses were defined individually for each tumor model to reach an estimated permanent local tumor control rate between 30–50% in the RCTx group. Therefore, total doses between 30 and 93 Gy in 30 fractions within 6 weeks were given. During each fraction, the animals were immobilized using plastic tubes fixed on a lucite plate with the tumor-bearing leg held in position by a foot holder distal to the tumor. Irradiations under normal blood flow conditions were given to unanesthetized air-breathing animals. When tumors reached a volume of at least 113.1 mm^3^ (corresponding to diameters of 6 × 6 mm), animals were randomly allocated into three different treatment groups. Measurements of tumor volumes before the first treatment intervention are summarized in Additional file 1: Table S1. In the control group, animals received RCTx and saline as vehicle. In the two intervention groups, nimorazole was applied with the first or after tenth fraction. At weekends, no treatment (irradiation, nimorazole or cisplatin) was administered. Furthermore, from each treatment group, 6–18 tumors were excised 24 h after the tenth fraction for immunohistochemistry. As control, 10–14 untreated tumors were excised per tumor model. For local tumor control and histological analysis, animals were excluded from the analysis if 10% of the scheduled fractions (3 out of 30 fractions and 1 out of 10 fractions respectively) or more were missed, i.e., because the leg was retracted during irradiation. The body weight of animals was determined once per week.

### Follow up

Tumor diameters were measured twice per week using a caliper for the first 90 days after irradiation and thereafter once per week. The tumor volume was calculated for each time point as _*π/*6 · *a* · *b*_^2^, where _*a*_ is the longest and _*b*_ is the perpendicular shorter tumor diameter. The animals were sacrificed when the recurrent tumor reached the diameter of 15 mm or when the animal appeared to suffer.

### Local tumor control

Local tumor control was evaluated until day 120–180 after the end of irradiation dependent on the tumor model, which is sufficient to detect virtually all tumor recurrences (Additional file 1: Table S2). Local failures were scored when the tumor volume increased monotonically within five measurements or strictly monotonically within three measurements after shrinkage, or when the tumor continued to grow without shrinkage. Increase (decrease) was defined as a relative change of at least 7% between two measurements, taking measurement inaccuracies into account. Censored animals were included in the analysis, when they had a follow-up for at least 20 days after the last fraction. Recurrences after 90 days were confirmed through histological evaluation. Kaplan–Meier estimates of tumor control rates from the different treatment groups are reported. Sample size to compare tumor control rates was estimated before the experiment using the method described in Machin et al. [31], which assumes a proportional hazard over time. Power analysis indicated that a minimum of 45 individual per arm would be needed to identify a difference of 30% in TCR, e.g., from 30 to 60%, assuming a power of 80% and a two-sided significance level (alpha) of 0.05. Supposing that tumor transplantation may fail in some cases, the experiment was conducted up to a maximum of 56 animals per group. The estimated samples size of 45 individuals was achieved in all tumor models expect for FaDu and UT5, where the dropout due to transplantation failure was higher (Additional file 1: Table S2).

### Histological study

A total of 32–44 tumors per model were used for histological analysis. Animals were injected with the hypoxia marker pimonidazole (Natural Pharmacia International, Inc., Research Triangle Park, NC, USA; 0.1 mg/g b.w., dissolved at 10 mg/ml in 0.9% NaCl, i.p.) one hour before excision and with the perfusion marker Hoechst 33342 (Sigma Aldrich, Deisenhofen, Germany; 0.75 mg in PBS, intravenously [i.v.]) one minute before excision. The tumor was immediately snap frozen in liquid nitrogen and stored at -80 °C. Up to three 10 µm frozen cross-sections from the center of the tumor with a distance of 70 µm were stained for pimonidazole (rabbit antipimonidazole antisera, Burlington, USA) and CD31 (rat anti-mouse CD31, clone MEC 13.3, PharMingen/BD Biosciences, Heidelberg, Germany), scanned and blindly analyzed as described previously [5]. After scanning, the same tumor sections were stained with haematoxylin and eosin for identification of viable and necrotic tumor subareas. To avoid bias, the threshold values were defined by the same person (L.K.). The pimonidazole hypoxic fraction and the relative vascular area were calculated as the percentage of the viable tumor area stained for pimonidazole or CD31, respectively. The pimonidazole hypoxic volume, as a surrogate of the number of hypoxic cells, was calculated as a product of the pimonidazole positive area relative to the total tumor area and tumor volume at time of excision. The fraction of perfused vessels was calculated as the percentage of the vascular area overlapping with Hoechst 33342 signal in the viable tumor subarea. Necrotic fraction was determined as the necrotic tumor area divided by the total tumor area. For statistical analysis, mean values of up to three sections from each tumor were determined to calculate all histological parameters. Each experimental or control group included 9 to 16 tumors.

### RNA-profiling

For RNA-profiling, 10 µm frozen cross-sections of untreated tumors and tumors after 10fx RCTx with and without nimorazole were used. Per tumor model and treatment, five individual tumors were used and total RNA (80 ng) were extracted according to the manufacturer’s instructions (Qiagen, RNeasy Mini Kit). Quality and purity were determined using the Qubit fluorometer (Life Technologies GmbH). Gene expression analyses were performed using nanoString technologies as described previously [32, 33]. Briefly, the nanoString panel comprised 209 genes, including two hypoxia gene signatures (Toustrup et al. [9], Eustace et al. [10]), as well as potential stem cell markers *MET*, *SLC3A2*, and *CD44*.

### Validation in patient cohort

Differentially expressed genes in xenograft models were validated in an independent patient cohort investigated and provided by the German Cancer Consortium—Radiation Oncology Group (DKTK-ROG) [33]. Briefly, 158 patients with locally advanced HNSCC received primary RCTx based on cisplatin (81.6%) or mitomycinC (18.4%) between 2005 and 2011 (details described in [33]). For 137 out of 158 patients, gene expression profiling has been performed before treatment using the Affymetrix HTA2.0 platform. Kaplan–Meier estimates and multivariable Cox proportional hazards models are reported.

### Statistical analysis

All analyses were conducted using R (4.3.1) and the following packages: DGE analysis was performed using limma (3.56.1) [34]. Preprocessing of the microarray data was performed using oligo (1.56.0) and biomaRt (2.48.3). For log-rank tests, Cox regression and corresponding plots, the survival (3.5–5), multcomp (1.4–23), and survminer (0.4.9) packages were utilized. Plots were created either using ggplot2 (3.4.2) or ComplexHeatmap (2.16.0). Two data scientists (V.B., S.L.), as part of our team, performed the statistical analysis.

#### Local tumor control

The evaluation of tumor control rates were conducted via an automated script and reviewed afterwards (V.B., L.K.). To compare hazards among treatment groups, univariable Cox proportional hazards models were fit, after testing model assumptions. P values were corrected for multiple comparisons, (i.e., RCTx + nimorazole vs RCTx + carrier and RCTx + nimorazole after 10fx vs RCTx + carrier) by applying a Closed Dunnett procedure [35]. Adjusted values of p < 0.05 were considered statistically significant.

#### Histological evaluation

We used classical closed testing for all histological parameters [35], with the primary null hypothesis that the median measurements of all treatment groups are equal within one tumor model using the Kruskal–Wallis test. If the primary null hypothesis was rejected, pairwise Wilcoxon rank sum tests were conducted (Untreated vs 10fx RCTx + nimorazole, Untreated vs 10fx RCTx + carrier). Adjusted values of p < 0.05 were considered statistically significant. Comparisons were visualized using box plots following the standard Tukey representations. Boxes represent the interquartile range (IQR), with the horizontal line indicating the median value. Whiskers indicate the largest (respectively smallest) value within 1.5 times the IQR above the 75th (respectively below the 25th) percentile.

#### RNA-profiling

Raw counts of nanoString data were normalized by positive controls counts and housekeeping genes *ACTR3, B2M, GNB2L1, NDFIP1, POLR2A, RPL11, RPL37A*, as described by the manufacturer (nanoString, MAN-C0011-04, Gene Expression Data Analysis Guidelines), and logarithmized. For differential gene expression analysis, the mean expressions of individual tumor models were compared against each other (e.g., mean of RCTx + nimorazole-treated FaDu samples against mean of RCTx + nimorazole-treated SAT samples) instead of summarizing multiple tumor models (e.g., mean of all RCTx + nimorazole-treated responding models against mean of all RCTx + nimorazole-treated non-responding models). This prevents to bring up genes where only the mean of the summarized tumor models is significantly different to another group, but not the individual means of all tumor models. False discovery rate at 10% across all genes and group comparisons were controlled using the Benjamini and Hochberg method [36]. Comparisons were visualized using box plots as described in Histological evaluation.

#### Validation in patient cohort

Raw data was normalized using the Robust Multichip Average (RMA) method. For those genes containing multiple probes in the array, their median expression was used for further analysis. Patients were split into one of two groups according to DEG using k-means clustering based on the Euclidean distance. To compare LRC among these groups, Kaplan–Meier estimates and multivariable Cox proportional hazards models (after testing model assumptions) were fit. Reported p values of < 0.05 were considered statistically significant.

## Results

Both, RCTx and application of nimorazole were well tolerated by the animals. Only at the beginning of nimorazole treatment, a temporary elevated blood circulation of the skin, visible as slight redness, was observed. This effect was not detectable after later applications, which might be an adaption to the treatment. Overall, no relevant differences in body weight between treatment groups or tumor models were observed (Additional file 1: Fig. S1).

**Fig. 2:**
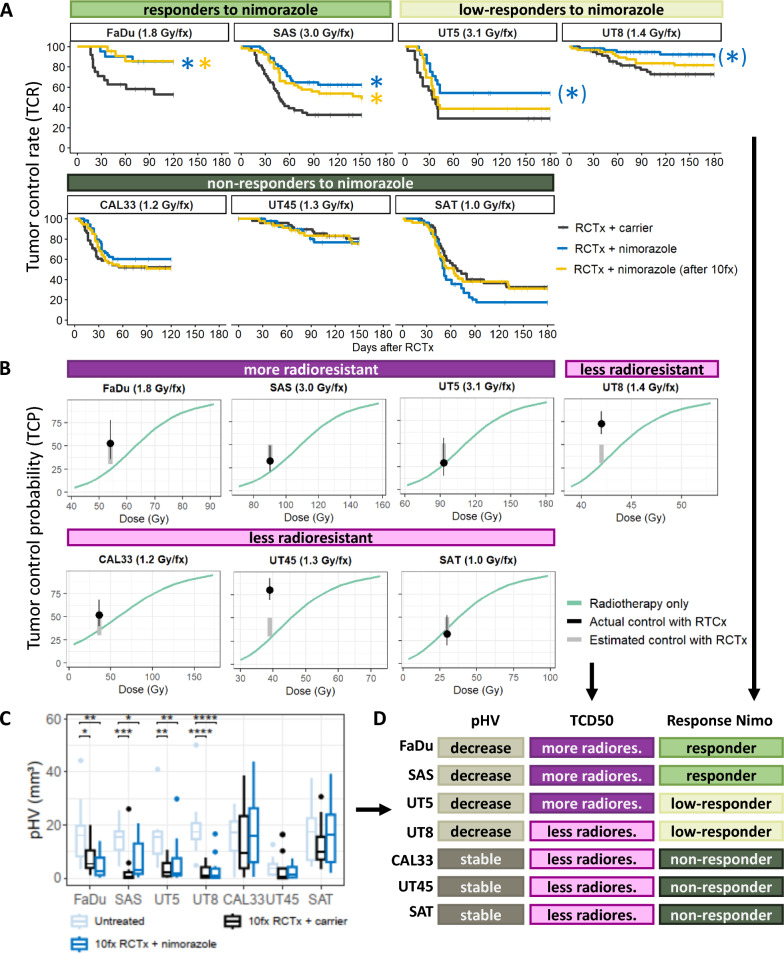
**A** Kaplan–Meier estimates of the seven tumor models after radiochemotherapy (RCTx) with 30 fractions in 6 weeks, weekly cisplatin and nimorazole or carrier. Curves significantly different from the RCTx + carrier curve are marked with an asterisk *. Responder models showed improved tumor control rate (TCR) in both nimorazole-treated arms, low-responder models showed a positive trend in TCR only when nimorazole was administered with the first fraction [marked with (*)], non-responder models showed no positive effect in neither nimorazole-treated arm. **B** Summarized tumor control probability (TCP) for every tumor model irradiated with 30 fractions in 6 weeks with radiotherapy only (green line) performed in previous experiments [6, 27–29]. Estimated radiation doses for tumor control rate of 30–50% for RCTx are shown as gray, bold line. Black lines visualize the actual tumor control rate with RCTx from Kaplan–Meier estimates (dot = estimate, line = 95% confidence interval). FaDu, SAS, UT5 were classified as more radioresistant, UT8, CAL33, UT45, SAT as less radioresistant based on TCD_50_ cutoff of 60 Gy. **C** Histological evaluation of the pimonidazole hypoxic volume (pHV) for the seven tumor models untreated (leftmost bars) and after RCTx with 10 fractions in 2 weeks combined with carrier (middle bars) or nimorazole (rightmost bars). The box plots displayed adhere to the Tukey style (see Methods). P value cutpoints: **** < 1e-04, *** < 0.001, ** < 0.01, * < 0.05. **D** Summary of the tumor models’ characteristics from (**A**–**C**), radioresistant abbreviated as radiores

The effect of nimorazole on tumor control rate (TCR) showed pronounced heterogeneity among the seven tumor models (Fig. 2A, Table 2a and b). Two models (FaDu and SAS) showed a significantly higher TCR in both nimorazole arms compared to RCTx alone. For two further models (UT8, UT5), the results indicated an increase in local tumor control when nimorazole was added starting with the first fraction of RCTx but differences in TCR are statistically non-significant after correcting for multiple testing. This suggests that both, UT5 and UT8, may benefit from adding nimorazole to radiochemotherapy but to a lower extent than FaDu or SAS. For CAL33, UT45 and SAT, no improvement of local tumor control for combined RCTx with nimorazole compared to RCTx alone was observed. Radiation doses in this study were prescribed individually per tumor model to reach, based on previous experiments [6, 27–29], an estimated local tumor control rate between 30–50% in the RCTx + carrier arm. Figure 2B highlights that the estimated and actual control rate match for most tumor models, except for UT8 and UT45, showing a more sensitive response to RCTx than expected. In general, more radioresistant tumors according to TCD_50_ values showed a more pronounced effect to the addition of nimorazole than less radioresistant ones. However, also radiosensitive (according to TCD_50_ values) UT8 showed the potential for an increase in TCR with nimorazole when administered with the first fraction.

**Table 2:** Local tumor control of tumor models. a) Tumor control rate (TCR) until day 120–180 after RCTx with 30 fractions in 6 weeks, weekly cisplatin and nimorazole or carrier. b) Hazard ratios (HR) and corresponding 95% confidence intervals of RCTx + nimorazole vs RCTx + carrier and RCTx + nimorazole (after 10fx) vs RCTx + carrier, p values after (p adj) correcting for multiple testing

Tumor model	Cumultative dose [Gy]	RCTx + nimorazole		RCTx + nimorazole (after 10fx)		RCTx + carrier	
		TCR [%]	[95% CI]	TCR [%]	[95% CI]	TCR [%]	[95% CI]
FaDu	54	85.0	[70.7, 100.0]	85.4	[71.4, 100.0]	52.8	[35.7, 77.9]
SAS	90	62.3	[50.2, 77.3]	48.0	[35.5, 64.8]	32.4	[21.4, 49.1]
UT5	93	54.2	[37.5, 78.3]	38.5	[23.7, 62.5]	29.0	[15.0, 55.9]
UT8	42	89.0	[80.2, 98.9]	81.4	[71.6, 92.5]	72.6	[61.3, 86.0]
CAL33	36	60.2	[48.3, 75.0]	50.8	[39.0, 66.3]	52.0	[39.8, 68.0]
UT45	39	76.5	[63.4, 92.4]	75.5	[62.2, 91.5]	80.2	[69.3, 92.8]
SAT	30	17.5	[9.4, 32.6]	31.0	[19.3, 50.0]	32.3	[19.9, 52.4]

Tumor model	RCTx + nimorazole			RCTx + nimorazole (after 10fx)		
	HR	[95% CI]	p adj	HR	[95% CI]	p adj
FaDu	0.24	[0.07, 0.88]	*0.043	0.22	[0.06, 0.81]	*0.043
SAS	0.37	[0.21, 0.66]	*0.002	0.54	[0.32, 0.91]	*0.022
UT5	0.44	[0.20, 0.96]	0.074	0.69	[0.34, 1.41]	0.311
UT8	0.32	[0.11, 0.88]	0.052	0.63	[0.28, 1.43]	0.270
CAL33	0.69	[0.38, 1.26]	0.376	0.91	[0.52, 1.60]	0.752
UT45	1.22	[0.47, 3.16]	0.872	1.23	[0.49, 3.10]	0.872
SAT	1.58	[0.97, 2.59]	0.119	1.17	[0.69, 1.98]	0.565

Significantly different HR compared to RCTx + carrier are marked with an asterisk *

Several histological parameters were investigated as putative biomarkers for the effect of nimorazole in addition to RCTx, i.e., pimonidazole hypoxic volume (pHV), pimonidazole hypoxic fraction (pHF), perfused fraction (PF), relative vascular area (RVA) and necrotic fraction (NF). Overall, the histological parameters of untreated tumors did not support the assumption of pre-treatment differences in hypoxia between models which show higher TCR when nimorazole is added to RCTx and the remaining models. With the exception of the pHV of UT45 (4.5 mm^3^), all pHV values ranged between 14.9 and 19.2 mm^3^ before treatment. Four models (FaDu, SAS, UT8, UT5) showed a statistically significant lower pHV after ten fractions of RCTx than their untreated counterparts (Fig. 2C). According to the Kaplan–Meier estimates (Fig. 2A), these models are also the most responsive to nimorazole addition to RCTx: The lower pHV was apparent in both RCTx arms, with and without nimorazole, indicating that the reduction of pHV is driven by the response to RCTx and not nimorazole. For CAL33, UT45 and SAT, in which the addition of nimorazole did not increase TCR compared to RCTx alone, no significant change of the pHV after 10 fractions was observed. Here, pHV remained on a similar level during treatment as in untreated samples (see also Additional file 1: Fig. S2). A reduction in the pHV can result from a reduction of the proportion of hypoxic cells within a tumor, a reduction of the overall tumor volume or both. After 10 fractions of RCTx (with and without nimorazole) none of the tumor models showed a significant lower tumor volume compared to untreated volumes (Additional file 1: Fig. S3A). Thus, our data indicate that the proportion of hypoxic cells was decreased by RCTx in FaDu, SAS, UT8, UT5, but not in CAL33, UT45 and SAT. The pimonidazole hypoxic fraction (pHF, Additional file 1: Fig. S3B) was smaller in FaDu and UT8 in both treatment arms, and for SAS in the 10fx RCTx + carrier arm compared to untreated samples. Only some small alterations were observed in PF (Additional file 1: Fig. S3C) and RVA (Additional file 1: Fig. S3D) in treated compared to untreated samples. Irradiation of the tumors led to a significantly higher NF in SAS (both treatment arms) and SAT (carrier arm) (Additional file 1: Fig. S3E). Taken together, out of the histological parameters studied, only the change in pHV after ten fractions of RCTx was associated with in an increase of TCR when nimorazole was added to RCTx (Fig. 2D).

**Fig. 3:**
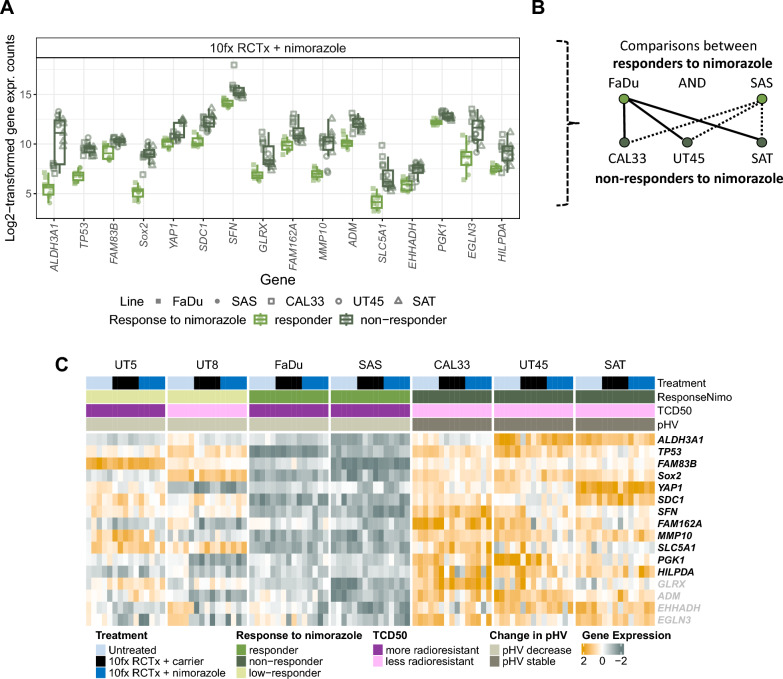
Results of RNA-Profiling. **A** Results of differential gene expression (DGE) analysis of responding (FaDu, SAS) and non-responding (UT45, CAL33, SAT) models to nimorazole in RCTx + nimorazole treated samples. The box plots displayed adhere to the Tukey style (see Methods). See also Additional file 1: Table S3. **B** Comparisons considered in DGE (e.g., FaDu vs SAT, FaDu vs CAL33, et cetera). UT5 and UT8, showing only low response to nimorazole according to TCR, have been excluded from this consideration. **C** Heatmap of differentially expressed genes (DEG) in all treatment groups. Genes shown with grey labels are only differentially expressed in RCTx + nimorazole treated samples but not in untreated samples. UT5 and UT8, not part of DEG analysis (left, grayed), illustrate a different pattern compared to responders and non-responders to nimorazole for the genes shown. Data is z-transformed, yellow: high expression, grey: low expression

For RNA-profiling, nimorazole-responding (FaDu, SAS) and non-responding models (UT45, CAL33, SAT) according to Fig. 2A were investigated. UT5 and UT8, representing a third, low-responding group, were excluded from the following analyses to avoid mitigating biological signals from clearly responding models. First, to identify genes that are influenced solely by the addition of nimorazole treatment, differential gene expression (DGE) analysis between 10fx RCTx + nimorazole and 10fx RCTx + carrier samples were conducted. While some differentially expressed genes (DEG) within individual models were found, none of them were shared among multiple models. Next, we investigated expression patterns between nimorazole-responding and non-responding models. Given the different degree of radioresistance (according to TCD_50_ values) of these tumor models, we first compared treated samples, in which the effects of radioresistance are mitigated by the individualized radiation doses. This enables to identify genes, which may be associated with the pronounced response to RCTx also on hypoxic cells in FaDu and SAS, as indicated by the significant lower pHV compared to untreated samples. DGE analysis revealed 16 genes being significantly upregulated in non-responders compared to responders (Fig. 3A and B, Additional file 1: Table S3) in RCTx + nimorazole treated samples. We then compared pre-treatment samples to test whether the observed differences were induced by the effect of radiochemotherapy. From 16 genes, 12 genes (*ALDH3A1, TP53, FAM83B, Sox2, YAP1, SDC1, SFN, FAM162A, MMP10, SLC5A1, PGK1, HILPDA*) expressed a distinct pattern also in pre-treatment samples, while the remaining four genes (*GLRX, ADM, EHHADH, EGLN3*) were different only in treated samples (Fig. 3C). Because we found no differences that can be ascribed to the addition of nimorazole alone, we presumed that the 12 genes may play a more general role in radiochemotherapy outcome and potentially predict tumor control. From the 12 genes, one gene (*FAM162A*) belongs to the hypoxia 15 gene signature by Toustrup et al. [9], while four more genes (*FAM83B, SDC1, PGK1, HILPDA*) are part of the hypoxia 26 gene signature by Eustace et al. [10]. According to these hypoxia gene signatures, more hypoxic tumors might be expected to express on average higher levels of those genes. However, in the tumor models investigated here, no clear pattern between responders and non-responders emerged for the previously published signatures (Additional file 1: Fig. S4A, B), neither before nor during treatment. Only UT8, a low-responding tumor model according to Fig. 2A, depicted a clear downregulation of hypoxia-related genes for both treatment arms. However, for the nimorazole-responding models FaDu and SAS, no difference was found.

**Fig. 4:**
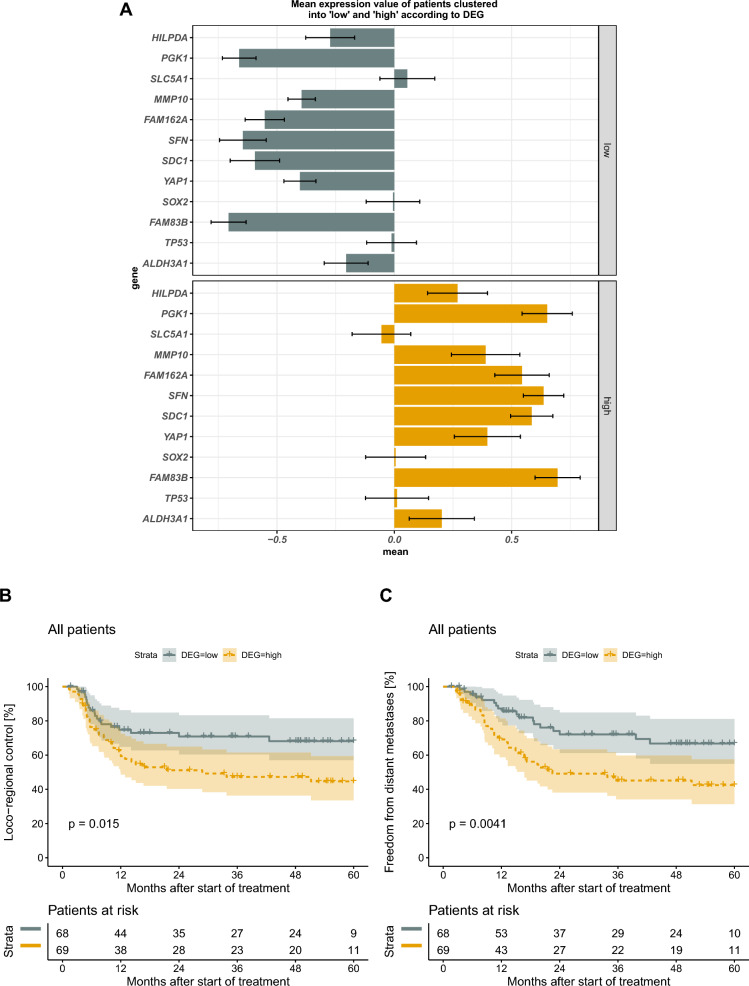
Validation on retrospective HNSCC cohort of the DKTK-ROG that received primary RCTx. **A** Patients are split into two groups according to differentially expressed genes (DEG). Patients with an overall lower expression are grouped into”low”, patients with an overall higher expression are grouped into”high” using k-means clustering. Data is z-transformed, error bars indicate standard error of the mean. **B**, **C** Kaplan-Meier estimates on loco-regional control and distant metastases, for patients grouped into low and high gene expression. P values correspond to log-rank tests.

We investigated whether the genes from DGE analysis from HSNCC xenografts are predictive for RCTx response in patients. In the retrospective DKTK-ROG cohort that received primary RCTx, patients received a comparable treatment protocol as the examined tumor models (without nimorazole) with LRC as primary endpoint and biopsies taken before treatment. We assumed that if the found genes are predictive for RCTx outcome, patients with an overall lower expression would show a superior LRC compared to patients with an overall higher expression profile. Derived from the results in our xenograft models, lower gene expression values might indicate also in patients the potential of RCTx to effectively diminish hypoxic volume. In total, 68 patients were assigned to the”low” and 69 patients to the”high” group (Fig. 4A). In line with our hypotheses, Kaplan–Meier estimates of LRC and distant metastases show a significantly increased risk for patients with higher expression profiles (Fig. 4B, C). Notably, individual genes were not able to split patients into two risk groups for LRC (Additional file 1: Fig. S5). Other patients’ characteristics were balanced among groups (Table 3), despite p16 status, a surrogate marker for HPV infection, i.e., significantly more p16 positive patients depicted only low expressions of the 12 genes. Correlation analysis between p16 status and our gene grouping revealed only weak associations (phi coefficient 0.2). As p16 positivity has shown to be associated with beneficial treatment outcome, multivariable Cox regression (included N stage, p16, log-transformed tumor volume and DEG grouping) was performed (Table 4). In multivariable analysis, patients with p16-negative tumors and high expressions for the 12 DEG were associated with higher risk for loco-regional failure (HR 3.44 [1.06, 11.24]) and HR 1.81 [1.00, 3.26] respectively). Taken together, the DEG found were able to split patients with HNSCC into two risk groups for RCTx response with LRC as endpoint.

**Table 3:** Validation on retrospective HNSCC cohort of the DKTK-ROG that received primary RCTx. Group characteristics when patients are split according to differentially expressed genes (DEG) into low and high expression groups

	Level	Low	High	p
n		68	69	
Gender (%)	f	15 (22.1)	10 (14.5)	0.355
	m	53 (77.9)	59 (85.5)	
Age (mean [SD])		58.90 (9.44)	58.64 (9.51)	0.873
Chemotherapy (%)	Cisplatin	58 (85.3)	56 (81.2)	0.675
	Mitomycin C	10 (14.7)	13 (18.8)	
p16 (%)	Positive	15 (22.1)	6 (8.7)	*0.040
	Negative	47 (69.1)	60 (87.0)	
	(Missing)	6 (8.8)	3 (4.3)	
HPV16 (%)	Positive	12 (17.6)	4 (5.8)	0.054
	Negative	55 (80.9)	65 (94.2)	
	(Missing)	1 (1.5)	0 (0.0)	
T stage (%)	T2	12 (17.6)	5 (7.2)	0.161
	T3	19 (27.9)	19 (27.5)	
	T4	37 (54.4)	45 (65.2)	
N stage (%)	N0	9 (13.2)	12 (17.4)	0.296
	N1	2 (2.9)	3 (4.3)	
	N2	8 (11.8)	6 (8.7)	
	N2a	4 (5.9)	10 (14.5)	
	N2b	21 (30.9)	14 (20.3)	
	N2c	18 (26.5)	22 (31.9)	
	N3	6 (8.8)	2 (2.9)	
UICC stage (%)	III	6 (8.8)	6 (8.7)	1.000
	IV	62 (91.2)	63 (91.3)	
Tumor localization (%)	Oral cavity	8 (11.8)	14 (20.3)	0.507
	Oropharynx	35 (51.5)	29 (42.0)	
	Hypopharynx	20 (29.4)	18 (26.1)	
	Oral cavity / Oropharynx	2 (2.9)	1 (1.4)	
	Oropharynx / Hypopharynx	2 (2.9)	4 (5.8)	
	Oral cavity / Oropharynx / Hypopharynx	1 (1.5)	3 (4.3)	
ln(GTV) (mean [SD])		3.11 (0.78)	3.34 (0.85)	0.098
DEG (%)	Low	68 (100.0)	0 (0.0)	< 0.001
	High	0 (0.0)	69 (100.0)	

Characteristics significantly different are marked with an asterisk *

For all categorical variables a Pearson’s Chi-squared test was performed, for all continuous variables [Age and ln(GTV)] an unpaired two-sample *t*-test (expecting equal variance) was performed

**Table 4:** Validation on retrospective HNSCC cohort of the DKTK-ROG that received primary RCTx. Multivariable Cox regression for loco-regional control

Loco-regional control			
Parameter	HR	[95% CI]	p
p16 [negative vs positive]	3.44	[1.06, 11.24]	*0.0405
ln(GTV)	1.31	[0.94, 1.83]	0.1116
N stage [ordinal N0 to N3]	1.12	[0.95, 1.33]	0.1732
DEG [high vs low]	1.81	[1.00, 3.26]	*0.0499

*HR* hazard ratio, *95% CI* 95% confidence interval

N stage ranging from N0 to N3, p values considered as statistically significant are marked with an asterisk *

## Discussion

Our pre-clinical trial on HNSCC xenografts investigated the effect of the hypoxic cell radiosensitizer nimorazole on local tumor control after fractionated RCTx and potential prognostic biomarkers for the efficacy of nimorazole. The seven tumor models used here have been chosen to account for heterogeneity of the treatment response of HNSCC. Differences in response to fractionated RT are corroborated by the TCD_50_ values of the models, which were derived from previous experiments (Fig. 2B). Tumor hypoxia is one of the factors influencing radiation response to fractionated radiotherapy [37] and has previously been shown by our group to impact differences in TCD_50_ between different HNSCC xenografts including models investigated here [5, 6, 11, 27]. In our present study, we observed differences in efficacy of nimorazole when added to fractionated RCTx in the different tumor models. Heterogeneity in tumor hypoxia might contribute to this observation. In a clinical trial, predominantly patients having more hypoxic tumors showed improved LRC from the addition of nimorazole to RT compared to RT only [9]. For patients with less hypoxic tumors, treatment de-escalation using RT or RCTx alone (without nimorazole) is under investigation (DAHANCA 30, NCT02661152). In our study, with the exception of UT45, pre-treatment differences in hypoxia measured as pHV between tumor models were minor, and thus cannot explain for differences in nimorazole response. However, differences in residual pHV among tumor models became clearly apparent during RCTx with and without nimorazole (Fig. 2C). Interestingly, those four tumor models in which the pHV decreased after 10 fractions, showed an increase of TCR when nimorazole was added to RCTx (Fig. 2A). From this observation it may be hypothesized that nimorazole is effective to increase tumor control compared to RCTx alone preferentially in those tumors in which hypoxia is decreased already early during the course of RCTx.

In our experiments, different doses of fractionated irradiation were used to account for the differences in radioresistance between the tumor models and to achieve comparable local tumor control rates of approximately 30–50%. Those four tumor models, which were irritated with higher doses (1.4 Gy to 3.1 Gy per fraction) compared to the other three models (1.0 Gy to 1.3 Gy per fraction), are also those which showed a significant decrease in pHV. Therefore, it cannot be excluded that the reduction in pHV observed in the four tumor models, does not reflect differences in tumor biology but rather that higher doses of radiation were more effective at reducing pHV. Such an effect might be mediated by more pronounced tumor regression after higher doses leading to a more pronounced decrease in pHV. However, this was not observed in our study as none of the tumor models showed a significant lower tumor volume after 10 fractions compared to untreated volumes. Also, residual hypoxia measured as pHV after 10 fractions with a uniform dose of 2 Gy in six HNSCC xenografts models was associated with TCD_50_ after local tumor control in a previous study [6]. A prognostic association of pHV and LRC has also been found in a clinical trial assessing residual hypoxia in patients with HNSCC undergoing RCTx using FMISO-PET [14, 16]. Taken together, determination of hypoxia early during treatment may have potential as a predictor for both, outcome of radio (chemo)therapy alone (as indicated by previous studies) and the effectiveness of addition of nimorazole. Nevertheless, further experiments are warranted to discriminate the relative impact of radiation dose versus biological determinants on the decrease of tumor hypoxia and to verify whether the pHV during RCTx qualifies as biomarker for an additional effect of nimorazole.

Comparing our two pimonidazole metrics, we see higher statistical evidence in using the pHV over the pHF as prognostic marker. Also, the pHV is arguably a more direct surrogate of the total number of hypoxic and thus radioresistant cells that need to be inactivated by radio(chemo)therapy for obtaining local tumor control than the pHF. This is supported by previous studies where the pHV was determined using different techniques, i.e., the Eppendorf histograph to assess the oxygen status of tumors together with computer tomography to estimate tumor volumes [38].

Besides measurements of hypoxia proportions, estimations of the vascular supply may explain treatment effects. It is known that the accumulation of anticancer drugs in solid tumors depends on vascularization, vessel permeability and the interstitial pressure [39]. Dependent on the distance of hypoxic cells to perfused areas, the capacity of agents like cisplatin or monoclonal antibodies to target hypoxic cells may be limited [40]. In our experiment, treatment effects on vascularization were negligible, i.e., differences in PF and RVA between untreated and treated samples were minor.

UT45, being the only HPV33 positive tumor model among our xenografts, represents a special case. It has been shown that HPV positive cells possess a higher intrinsic radiation sensitivity than HPV negative cells [41]. Contrary to patients with HPV-negative tumors, patients with HPV-positive tumors did not benefit from the addition of nimorazole to RT in the DAHANCA 5 trial [42], though HPV-positivity represents an independent, positive prognostic factor for LRC [33, 43]. In general, the overall higher intrinsic radiation sensitivity in HPV-positive tumors is not directly linked to a lower proportion of hypoxic cells [43, 44]. Hence, we decided for this study to investigate also the effects of nimorazole combined with RCTx on a HPV positive tumor model. In line with the clinical observations on RT alone, addition of nimorazole did not increase the effect of RCTx in UT45 tumors. However, it may also be hypothesized, that the sensitivity of this tumor model was already high at doses of 1.3 Gy/fx (TCR of 76.5% [63.4%–92.4%] at day 150 after RCTx) and its pre-treatment pHV (4.5 mm^3^) sufficiently low that no further sensitization through nimorazole was feasible. This is also supported by the median pHV during treatment (Fig. 2C), which is lower compared to untreated UT45 samples but failed to reach statistical significance.

Independent of tumor micromilieu parameters, also hypoxia gene signatures have proven to be prognostic in HNSCC on different endpoints [7–10]. Yet, in some independent HNSCC patient cohorts, where patients were treated with primary RCTx rather than RT alone, evidence for prognostic potential is lacking. For example, in the retrospective cohort of the DKTK-ROG that received primary RCTx, patients could not be stratified for LRC [33] by means of the gene signatures introduced by Lendhal et al. [45], Toustrup et al. [9], and Eustace et al. [10]. Further evaluations in an independent validation cohort yielded to similar, non-significant results, potentially limited by the small cohort size [46]. The prognostic value of the hypoxia 15 gene signature was also not confirmed for patients with oropharyngeal cancer treated with accelerated RCTx [47] and for patients recruited for the early closed trial on RCTx with nimorazole versus RCTx with placebo (DAHANCA 29-EORTC 1219 [25]). Overall, these findings suggest that existing hypoxia gene signatures may miss clinically relevant aspects of hypoxia in RCTx regimes. These results may also emphasize the need for reconsidering the time of hypoxia assessment, i.e. estimating hypoxia repeatedly during treatment instead of a single pre-treatment estimation. In our study, the gene signatures of Toustrup et al. and Eustace et al. did not support a difference in hypoxia among responders and non-responders to nimorazole (according to Fig. 2A), neither before treatment nor after 10 fractions. According to our analyses, these surrogate markers for hypoxia were not able to identify xenograft models eligible for nimorazole addition to RCTx in order to improve LRC. Therefore, we analyzed which genes differed in responding models to nimorazole (FaDu, SAS) and non-responding ones (CAL33, UT45, SAT). Notably, we found no DEG among multiple tumor models that could be ascribed to the addition of nimorazole only. However, we found several genes that discriminated responding and non-responding models to nimorazole in RCTx + nimorazole treated and pre-treatment samples. Five DEG, i.e., *FAM162A, FAM83B, SDC1, PGK1, HILPDA*, were associated with hypoxia already previously [9, 10]. The remaining genes *ALDH3A1, TP53, Sox2, YAP1, SFN, MMP10, SLC5A1*, are not known to be directly linked to tumor hypoxia. Instead, we hypothesize that they may indicate a relevant interplay of hypoxia and RCTx response. For example, Lee et al. demonstrated that patients with a high SOX2 protein expression were at significantly higher risk for recurrence than patients with a low expression [48]. In contrast, Chung et al. highlighted that high expressions of their derived Sox2 signature were significantly associated with favorable prognosis for overall survival and disease-free survival in patients with HNSCC [49]. Deraz et al. found that MMP-10 expression in patients with HNSCC, examined using immunohistochemistry, was significantly correlated with tumor invasiveness and metastasis [50]. Akervall et al. found increased YAP1 expression in pre-treatment biopsies of patients with HNSCC prognostic for short recurrence-free survival, short cause-specific survival and low RCTx response [51]. Because the DEG were upregulated already in untreated non-responder samples and we did not find evidence for genes that where differentially expressed solely due to the addition of nimorazole itself, we assumed that the identified genes rather indicate RCTx resistance per se than an effect of nimorazole. This is in line with our results, confirming that this gene expression profile is also relevant in humans by demonstrating a significant association with LRC in patients with HNSCC treated with RCTx. Expression levels of individual genes were not prognostic for LRC, suggesting a complex interplay of gene regulations and treatment response. In our experiments with xenografts, those models which expressed low degrees of the 12 genes were also those which showed a pronounced increase of TCR with the addition of nimorazole compared to RCTx alone. Based on these pre-clinical results, we hypothesize that patients with low expression profiles of the 12 genes qualify as candidates for nimorazole addition to RCTx. This question would be of interest to be further addressed on clinical materials of patients treated with RCTx and nimorazole. Other known markers that are associated with radioresistance, e.g., cancer stem cell (CSC) markers like *CD44* or *SLC3A1*, did not show up during DGE analysis. While hypoxia gene signatures and CSC marker expressions showed only week correlations in the past [33], hypoxia is known to contribute to CSC evolution [52]. Also, CSC markers were found to be an independent prognostic factor for LRC in the DKTK-ROG cohort (that received primary RCTx) previously [33] as well as in an independent validation cohort [46]. Therefore, differences in CSC might also explain differences in radioresistance. However, in our pre-clinical study, differences in CSC markers between responding and non-responding models to nimorazole (according to Fig. 2A) were not apparent.

There are several limitations of the present study. First, micromilieu parameters and the response to fractionated RCTx could not be determined in the same individual tumor, but parameters for a group of tumors were compared. These tumors originated from the same cryostock with the same genetic background. Second, radiation doses vary among tumor models to adjust for their difference in radiosensitivity. We aimed for comparable TCRs of about 30–50% in all tumor models after RCTx alone to be able to address the question of an additional nimorazole effect with comparable statistical rigor. For comparison, applying a high dose per fraction (e.g., 3.0 Gy) to all tumor models, might lead to very high tumor control rates in the RCTx arm in less radioresistant models (according to TCD_50_ values), such that no further improvement with the addition of nimorazole would be statistically verifiable despite the already comprehensive sample size. Applying a low dose per fraction (e.g., 1.0 Gy) to all tumor models would drop tumor control of more radioresistant models close to zero, such that the tumor volume would continue to increase even during treatment. In addition, we aimed for a constant overall treatment time in all models, to exclude the confounding heterogeneous impact of the so-called time factor of fractionated irradiation on tumor control [53]. Therefore, we changed the doses per fraction according to the expected tumor control probabilities. This impedes direct comparability of Kaplan–Meier estimates between the tumor models. As it was hypothesized in the past that lower radiation doses per fraction decrease the enhancement ratio (ER) of radiosensitizers [54], the effect of nimorazole in models treated with low doses per fraction could have been hampered by our experimental approach. However, that hypoxic cell radiosensitizers can be effective also at low doses was demonstrated by a study involving isometronidazole combined with fractionated irradiation (30 fractions in 6 weeks at doses of 1.1–1.2 Gy), which improved tumor control significantly in FaDu xenografts compared to irradiation only [55]. This is in line with an in vitro study on chinese hamster ovary fibroblasts cells, showing that also nimorazole can be an effective sensitizer at low radiation doses (0–4 Gy) with a stable ER at various drug concentrations and independent of radiation doses [56]. Furthermore, in our present study, UT8 (irradiated with 1.4 Gy/fx) suggested an improved tumor control when nimorazole application started concomitantly with RCTx. Another limitation is that the number of genes for DGE analysis was limited by the targeted panel to a total of 209 genes. Thus, we intend to do a more exhaustive comparison between gene expression profiles of RCTx treatments with and without nimorazole in the future. Also, we plan to refine and validate the DEG on further cohorts to identify which genes contribute most to tumor control. For example, for the DKTK cohort investigated here, differences in *Sox2* expression between patients clustered into the”high” and”low” group were negligible (Fig. 4A). However, in order to prevent overfitting and conclusions being drawn from one specific cohort, we plan to examine the genes on larger cohorts and study their molecular pathways further, before discarding specific gene candidates. In particular, we want to analyze if higher expressed gene profiles are associated with an impaired effect of RCTx on hypoxic cells by comparing (residual) hypoxic volumes in patient cohorts.

## Conclusions

To the best of our knowledge, our pre-clinical study is the first that provides insights into the effectiveness of nimorazole combined with primary RCTx and not just RT. Our results indicate that nimorazole can improve local tumor control in hypoxic tumors, with pronounced heterogeneity between different tumor models. More specifically, we identified three response groups to nimorazole combined with RCTx (i.e., responders, low-responders and non-responders). The change in pHV during RCTx showed promise as potential biomarker for an additional effect of nimorazole, but requires further investigations. Additionally, genes derived from HNSCC xenograft models were highlighted that were predictive for LRC in patients with HNSCC treated with RCTx. These genes may potentially contribute to identify patients eligible for a combinational treatment of nimorazole and RCTx to further improve LRC. On a more general scale, we were able to demonstrate that gene expression profiles of xenograft models can be translated to clinically relevant findings in cancer patients.

### Supplementary Information

**Additional file 1:** **Figure S1.** Rolling mean relative body weight of all tumor models over time of experiment, starting from the first treatment (day = 0). Vertical line represents approximate time point at which treatments were finished and follow-up measurements were carried out. **Figure S2.** Pseudo-colored images of representative sections from SAS (responder to nimorazole addition) and CAL33 (non-responder to nimorazole addition) tumors untreated and after 10fx RCTx treated with nimorazole. Green: hypoxia, pimonidazole; blue: perfusion, Hoechst 33342; red: vascular endothelium, CD31; grey necrotic area. Nimorazole abbreviated as nimo. **Figure S3.** Histological evaluation of (A) tumor volume (B) pimonidazole hypoxic fraction (pHF), (C) perfused fraction (PF), (D) relative vascular area (RVA) and (E) necrotic fraction (NF) and for seven different tumor models, untreated (leftmost bars) and after RCTx with 10 fractions in 2 weeks and cisplatin in combination with carrier (middle bars) or nimorazole (rightmost bars). The box plots displayed adhere to the Tukey style (see Methods). P value cutpoints: **** < 1e-04, *** < 0.001, ** < 0.01, * < 0.05. **Figure S4.** Hypoxia estimation using previously published gene signatures. (A) Expression values of hypoxia 15-gene signature. Only two genes (*ADM, FAM162A*) emerged in differential gene expression (DGE) analysis to be significantly different between responding (FaDu, SAS) and non-responding (UT45, CAL33, SAT) models to nimorazole. Of note, *Lox* is expressed inversely to other genes among responders and non-responders to nimorazole addition. Shown are only RCTx + nimorazole samples. The box plots displayed adhere to the Tukey style (see Methods). (B) Heatmap analysis of hypoxia 15 and hypoxia 26 gene signature on all treatment arms for individual tumor models. No clear expression pattern among responding, low-responding and non-responding models to nimorazole addition emerged for hypoxia-related genes. Only UT8 (low-responder to nimorazole addition) expressed a clear downregulation of genes from the two hypoxia gene signatures for both, RCTx + nimorazole and RCTx + carrier arm. Data is z-transformed, yellow: high expression, grey: low expression. **Figure S5.** Kaplan–Meier estimates of loco-regional control on retrospective HNSCC cohort of the DKTK-ROG that received primary RCTx. Patients are split according to their gene expression into one of two groups. Patients with gene expression higher than gene’s mean expression are categorized into”high”, patients with gene expression lower or equal to gene’s mean expression are categorized into”low”. Individual genes belong to differentially expressed genes (DEG), p values corresponds to log-rank test and were not adjusted for multiple testing. **Table S1.** Start mean tumor volume and corresponding 95% confidence intervals for the seven different tumor models and their assigned treatment group. Mean tumor volumes were calculated before animals received the first treatment. **Table S2.** Follow-up information for the seven different tumor models irradiated with fractionated irradiation within 6 weeks in combination with cisplatin and nimorazole or carrier. **Table S3.** Results of differential gene expression (DGE) analysis between nimorazole-responding and non-responding tumor models to nimorazole addition in RCTx + nimorazole treated samples. Shown are estimates of the log2-fold-changes per contrast. Genes are ranked in descending order according to their adjusted p value (all p. adj. < 0.001).

## Data Availability

The data generated and analyzed from xenograft models during the current study are available via Open Science Framework, including a documentation for data pre-processing and downstream analysis to ensure reproducibility of results. Access permissions will be granted to the scientific community by contacting the corresponding author and completing of a material transfer agreement. For data from the retrospective cohort of the DKTK-ROG, the authors kindly ask to contact the corresponding authors of Linge et al. [33].

## Abbreviations

[18F]-HX4: 18F-Flortanidazole
ARRIVE: Animal research: reporting of in vivo experiments
CI: confidence interval
DAHANCA: Danish Head and Neck Cancer Group
DEG: Differentially expressed genes
DGE: Differential gene expression
DKTK-ROG: German Cancer Consortium Radiation Oncology Group
ER: Enhancement ratio
FAZA: ^18^F-Fluoroazomycin-arabinoside
FMISO: F-Fluoromisonidazole
HPV: Human papillomavirus
HNSCC: Head and neck squamous cell carcinoma
HR: Hazard ratio
LRC: Loco-regional control
NF: Necrotic fraction
PET: Positron emission tomography
PF: Perfused fraction
pHF: Pimonidazole hypoxic fraction
pHV: Pimonidazole hypoxic volume
PMH: Princess Margaret Hospital Cancer Centre
RCTx: Radiochemotherapy
RMA: Robust multichip average
RT: Radiotherapy
RVA: Relative vascular area
TCP: Tumor control probability
TCR: Tumor control rate
