# Supplementary material for: Prognostic biomarkers for the response to the radiosensitizer nimorazole combined with RCTx: a pre-clinical trial in HNSCC xenografts
Source: J Transl Med. 2023 Aug 26;21:576. doi: 10.1186/s12967-023-04439-2 (PMC10464469; doi:10.1186/s12967-023-04439-2)
Supplement: Supplementary file 1 — Additional file 1: Figure S1. Rolling mean relative body weight of all tumor models over time of experiment, starting from the first treatment (day = 0). Vertical line represents approximate time point at which treatments were finished and follow-up measurements were carried out. Figure S2. Pseudo-colored images of representative sections from SAS (responder to nimorazole addition) and CAL33 (non-responder to nimorazole addition) tumors untreated and after 10fx RCTx treated with nimorazole. Green: hypoxia, pimonidazole; blue: perfusion, Hoechst 33342; red: vascular endothelium, CD31; grey necrotic area. Nimorazole abbreviated as nimo. Figure S3. Histological evaluation of (A) tumor volume (B) pimonidazole hypoxic fraction (pHF), (C) perfused fraction (PF), (D) relative vascular area (RVA) and (E) necrotic fraction (NF) and for seven different tumor models, untreated (leftmost bars) and after RCTx with 10 fractions in 2 weeks and cisplatin in combination with carrier (middle bars) or nimorazole (rightmost bars). The box plots displayed adhere to the Tukey style (see Methods). P value cutpoints: **** < 1e-04, *** < 0.001, ** < 0.01, * < 0.05. Figure S4. Hypoxia estimation using previously published gene signatures. (A) Expression values of hypoxia 15-gene signature. Only two genes (ADM, FAM162A) emerged in differential gene expression (DGE) analysis to be significantly different between responding (FaDu, SAS) and non-responding (UT45, CAL33, SAT) models to nimorazole. Of note, Lox is expressed inversely to other genes among responders and non-responders to nimorazole addition. Shown are only RCTx + nimorazole samples. The box plots displayed adhere to the Tukey style (see Methods). (B) Heatmap analysis of hypoxia 15 and hypoxia 26 gene signature on all treatment arms for individual tumor models. No clear expression pattern among responding, low-responding and non-responding models to nimorazole addition emerged for hypoxia-related genes. Only UT8 (low-responder to nim [file 12967_2023_4439_MOESM1_ESM.pdf]

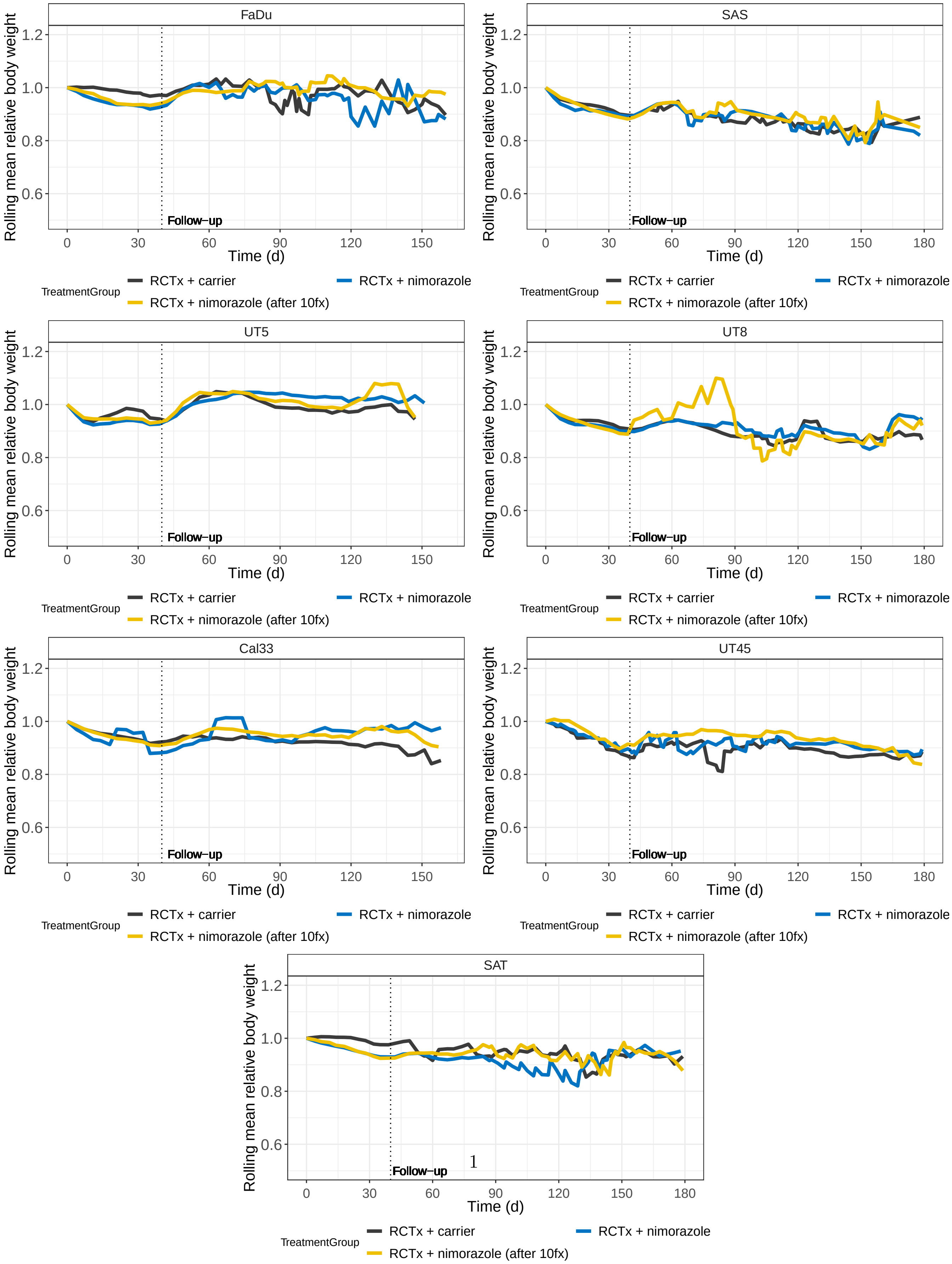
FaDu
SAS
UT5
UT8
Cal33
UT45
SAT
Rolling mean relative body weight
1.2
1.0
0.8
0.6
Follow−up
0
30
60
90
120
150
180
Time (d)
TreatmentGroup
RCTx + carrier
RCTx + nimorazole
RCTx + nimorazole (after 10fx)

Fig. S1

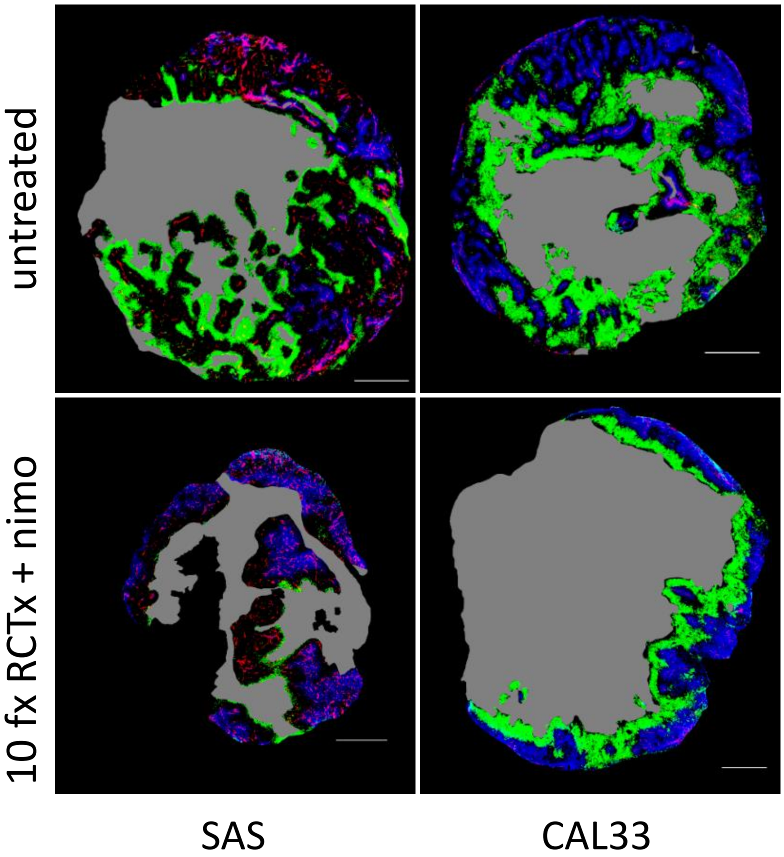

Fig. S2

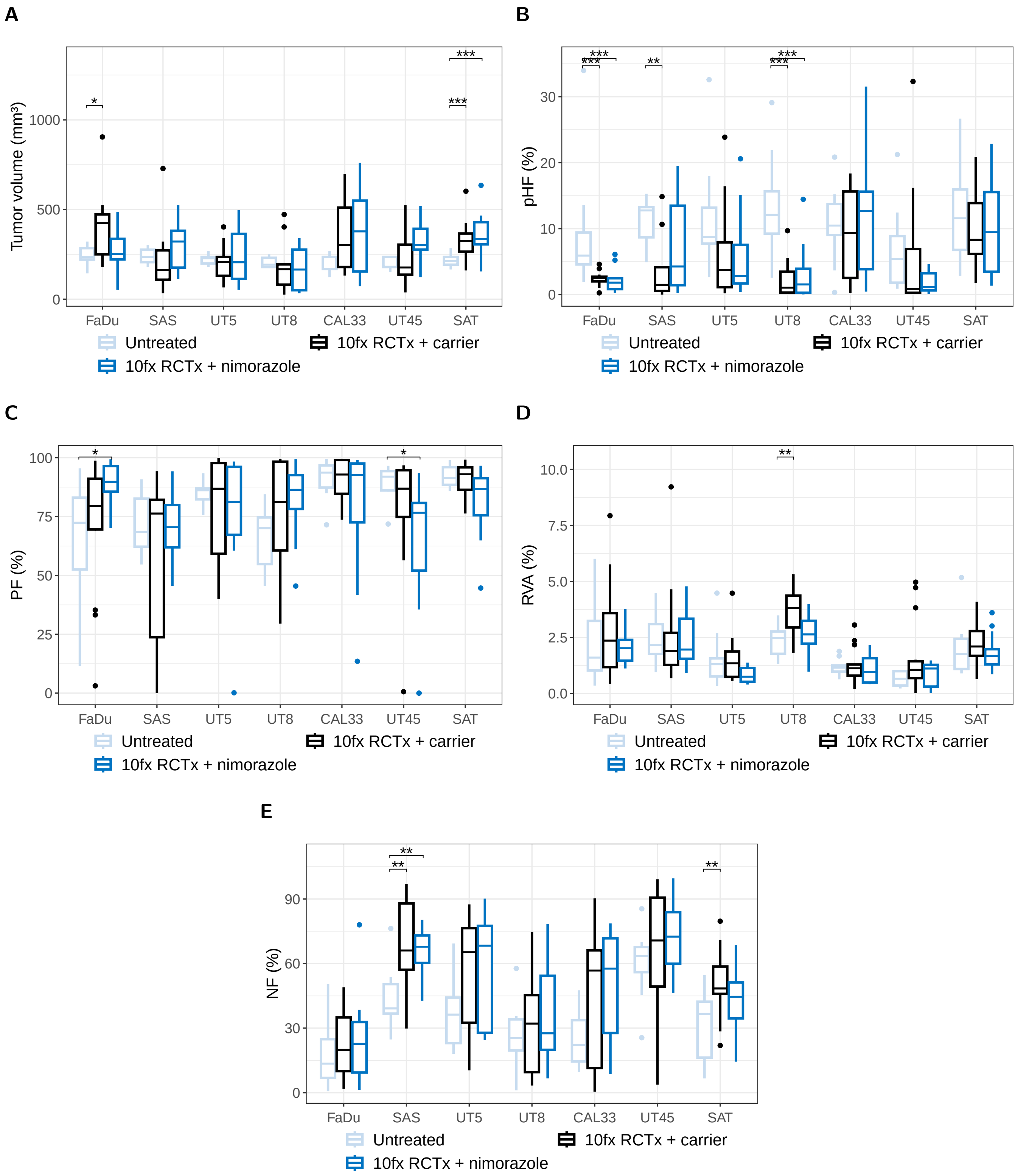
A
B
C
D
E
Tumor volume (mm³)
pHF (%)
PF (%)
RVA (%)
NF (%)
FaDu
SAS
UT5
UT8
CAL33
UT45
SAT
Untreated
10fx RCTx + carrier
10fx RCTx + nimorazole

Fig. S3

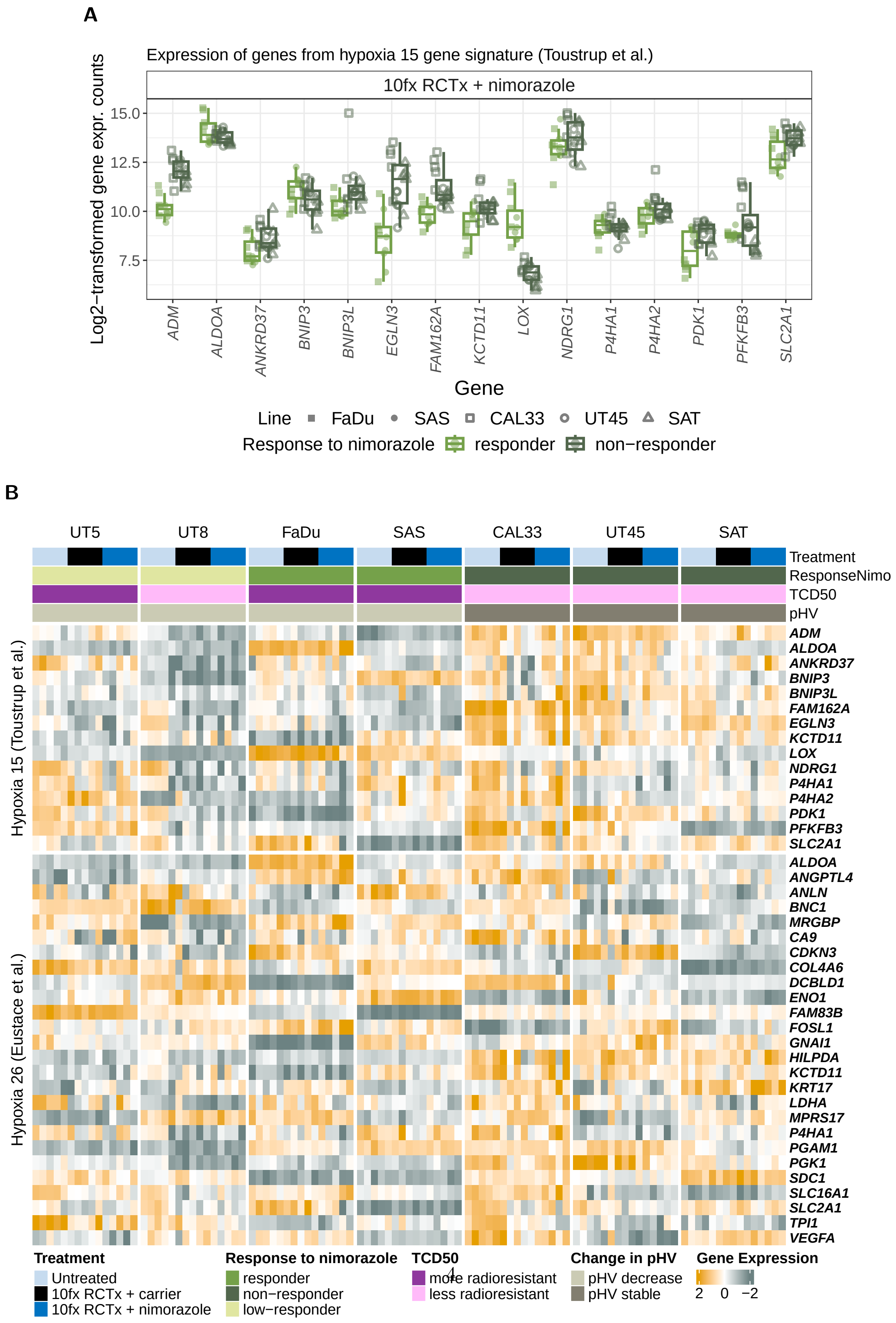
A
Expression of genes from hypoxia 15 gene signature (Toustrup et al.)
10fx RCTx + nimorazole
Log2−transformed gene expr. counts
15.0
12.5
10.0
7.5
ADM
ALDOA
ANKRD37
BNIP3
BNIP3L
EGLN3
FAM162A
KCTD11
LOX
NDRG1
P4HA1
P4HA2
PDK1
PFKFB3
SLC2A1
Gene
Line FaDu SAS CAL33 UT45 SAT
Response to nimorazole responder non−responder
B
UT5
UT8
FaDu
SAS
CAL33
UT45
SAT
Treatment
ResponseNimo
TCD50
pHV
Hypoxia 15 (Toustrup et al.)
Hypoxia 26 (Eustace et al.)
ADM
ALDOA
ANKRD37
BNIP3
BNIP3L
FAM162A
EGLN3
KCTD11
LOX
NDRG1
P4HA1
P4HA2
PDK1
PFKFB3
SLC2A1
ALDOA
ANGPTL4
ANLN
BNC1
MRGBP
CA9
CDKN3
COL4A6
DCBLD1
ENO1
FAM83B
FOSL1
GNAI1
HILPDA
KCTD11
KRT17
LDHA
MPRS17
P4HA1
PGAM1
PGK1
SDC1
SLC16A1
SLC2A1
TPI1
VEGFA
Treatment
Untreated
10fx RCTx + carrier
10fx RCTx + nimorazole
Response to nimorazole
responder
non−responder
low−responder
TCD50
more radioresistant
less radioresistant
Change in pHV
pHV decrease
pHV stable
Gene Expression
2 0 −2

Fig. S4

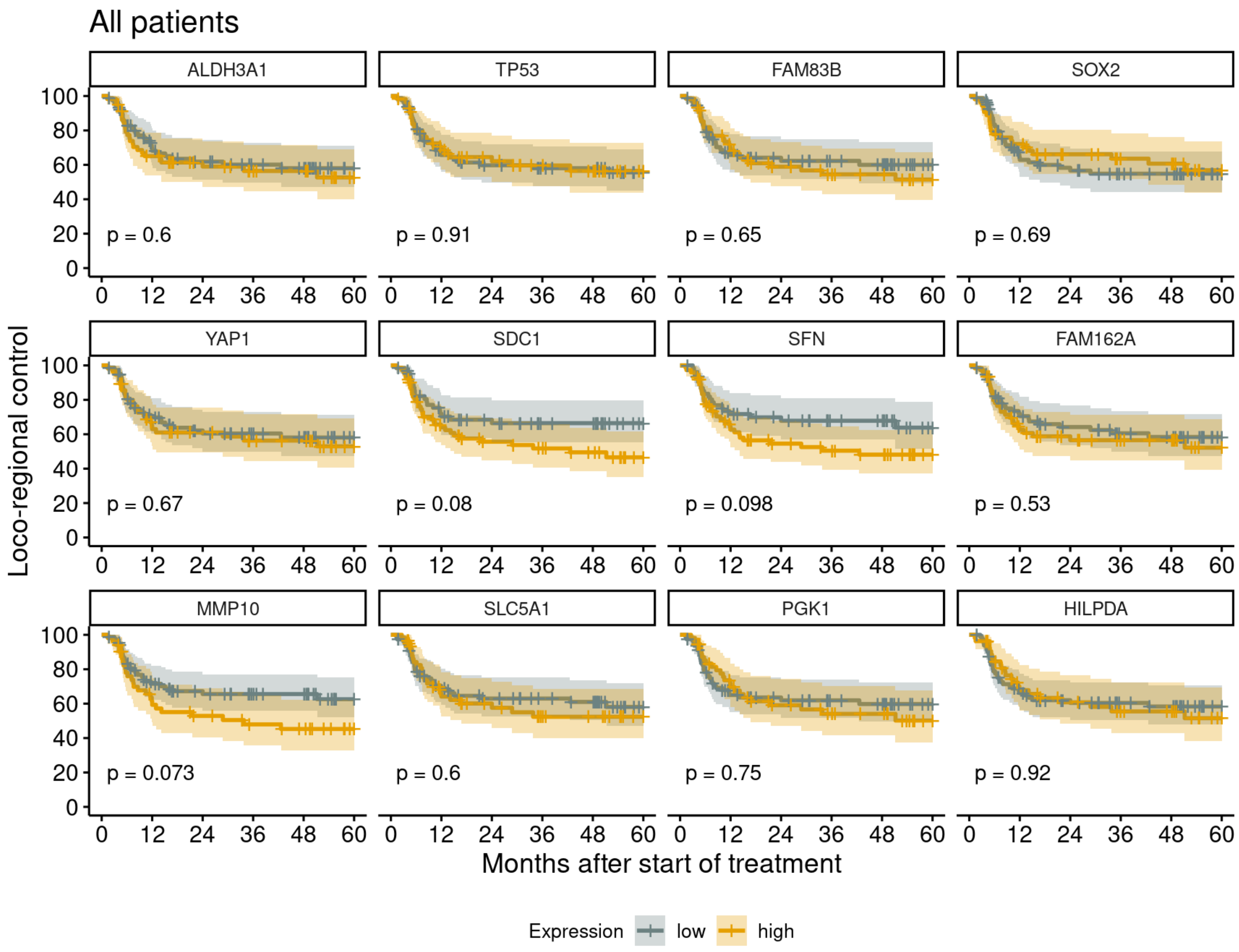
All patients
ALDH3A1
TP53
FAM83B
SOX2
p = 0.6
p = 0.91
p = 0.65
p = 0.69
YAP1
SDC1
SFN
FAM162A
p = 0.67
p = 0.08
p = 0.098
p = 0.53
MMP10
SLC5A1
PGK1
HILPDA
p = 0.073
p = 0.6
p = 0.75
p = 0.92
Loco-regional control
0 20 40 60 80 100
0 12 24 36 48 60
Months after start of treatment
Expression
low
high

Fig. S5

Table S1

Start mean tumor volume (mm^3) [95% CI]						
Tumor	RCTx + nimorazole		RCTx + nimo. (after 10fx)		RCTx + carrier	
FaDu	220.45	[202.05, 238.85]	201.28	[182.41, 220.14]	201.18	[176.98, 225.39]
SAS	221.71	[212.57, 230.84]	215.38	[205.85, 224.90]	232.73	[192.63, 272.83]
UT5	212.06	[199.75, 224.36]	197.74	[189.13, 206.35]	219.85	[205.60, 234.10]
UT8	199.63	[193.13, 206.14]	208.99	[200.07, 217.91]	207.36	[199.59, 215.13]
CAL33	197.82	[184.62, 211.02]	188.21	[176.98, 199.43]	197.60	[184.62, 210.59]
UT45	188.10	[179.25, 196.94]	185.80	[158.92, 212.68]	191.83	[184.41, 199.25]
SAT	205.45	[198.26, 212.64]	203.61	[194.98, 212.24]	207.32	[198.47, 216.17]

Table S2

	locally controlled tumors / total number of irradiated tumors			censored animals (censoring interval in days)			95% of all recurrences observed before day/last recurrence scored at day
Tumor	RCTx + nimorazole	RCTx + nimo. (after 10fx)	RCTx + carrier	RCTx + nimorazole	RCTx + nimo. (after 10fx)	RCTx + carrier	
FaDu	10/24	15/20	15/22	3/24 (54-85)	2/20 (76-83)	4/22 (42-107)	96/96
SAS	8/48	17/52	17/51	8/48 (48-139)	16/52 (36-139)	9/51 (36-146)	96/150
UT5	3/23	9/24	4/26	4/23 (38-169)	4/24 (85-169)	6/26 (85-176)	43/43
UT8	25/55	29/56	32/56	16/55 (26-176)	22/56 (31-166)	14/56 (27-167)	131/180
CAL33	17/51	26/53	23/53	10/51 (24-113)	6/53 (40-92)	4/53 (52-106)	48/90
UT45	27/50	19/43	19/45	14/50 (0-139)	16/43 (29-148)	17/45 (43-139)	138/141
SAT	6/47	6/54	6/51	14/47 (26-176)	6/54 (27-127)	15/51 (34-155)	92/132

Table S3

	FaDu.nimo - SAT.nimo	FaDu.nimo - CAL33.nimo	FaDu.nimo - UT45.nimo	SAS.nimo - SAT.nimo	SAS.nimo - CAL33.nimo	SAS.nimo - UT45.nimo
ALDH3A1	-4.89	-1.43	-6.34	-6.29	-2.83	-7.74
TP53	-2.65	-3.01	-3.54	-1.91	-2.27	-2.80
FAM83B	-0.57	-0.62	-0.43	-1.85	-1.91	-1.71
Sox2	-3.44	-3.13	-4.21	-3.85	-3.54	-4.62
YAP1	-2.05	-0.54	-0.41	-2.39	-0.87	-0.74
SDC1	-2.66	-2.02	-1.43	-2.55	-1.91	-1.32
SFN	-0.55	-1.77	-0.72	-1.07	-2.29	-1.24
GLRX	-0.69	-3.10	-1.11	-1.30	-3.71	-1.72
FAM162A	-0.62	-2.17	-0.61	-1.02	-2.57	-1.02
MMP10	-3.74	-3.24	-1.72	-3.81	-3.32	-1.80
ADM	-1.10	-1.71	-1.76	-1.75	-2.36	-2.41
SLC5A1	-1.29	-3.83	-1.75	-1.69	-4.23	-2.15
EHHADH	-1.70	-0.82	-1.61	-1.91	-1.04	-1.83
PGK1	-0.38	-0.92	-0.62	-0.34	-0.89	-0.59
EGLN3	-2.66	-3.59	-1.49	-3.22	-4.15	-2.05
HILPDA	-1.91	-2.10	-1.25	-1.64	-1.83	-0.97
